# Structural chromosome abnormalities, increased DNA strand breaks and DNA strand break repair deficiency in dermal fibroblasts from old female human donors

**DOI:** 10.18632/aging.100723

**Published:** 2015-02-04

**Authors:** Faiza Kalfalah, Sabine Seggewiß, Regina Walter, Julia Tigges, María Moreno-Villanueva, Alexander Bürkle, Sebastian Ohse, Hauke Busch, Melanie Boerries, Barbara Hildebrandt, Brigitte Royer-Pokora, Fritz Boege

**Affiliations:** ^1^ Institute of Clinical Chemistry and Laboratory Diagnostics, Heinrich-Heine-University, Med. Faculty, Düsseldorf, Germany; ^2^ Institute of Human Genetics and Anthropology, Heinrich-Heine-University, Med. Faculty, Düsseldorf, Germany; ^3^ Leibniz Research Institute for Environmental Medicine (IUF), Düsseldorf, Germany; ^4^ Molecular Toxicology Group, Dept. of Biology, University of Konstanz, Konstanz, Germany; ^5^ Systems Biology of the Cellular Microenvironment Group, Institute of Molecular Medicine and Cell Research, University of Freiburg, Freiburg, Germany; ^6^ German Cancer Research Centre (DKFZ), Heidelberg, Germany; ^7^ German Cancer Consortium (DKTK), Freiburg, Germany

**Keywords:** Aging, dermal fibroblasts, alkaline DNA unwinding, DNA double strand break repair, chromosome abnormalities, non-homologous end-joining

## Abstract

Dermal fibroblasts provide a paradigmatic model of cellular adaptation to long-term exogenous stress and ageing processes driven thereby. Here we addressed whether fibroblast ageing analysed *ex vivo* entails genome instability. Dermal fibroblasts from human female donors aged 20–67 years were studied in primary culture at low population doubling. Under these conditions, the incidence of replicative senescence and rates of age-correlated telomere shortening were insignificant. Genome-wide gene expression analysis revealed age-related impairment of mitosis, telomere and chromosome maintenance and induction of genes associated with DNA repair and non-homologous end-joining, most notably *XRCC4* and *ligase 4*. We observed an age-correlated drop in proliferative capacity and age-correlated increases in heterochromatin marks, structural chromosome abnormalities (deletions, translocations and chromatid breaks), DNA strand breaks and histone H2AX-phosphorylation. In a third of the cells from old and middle-aged donors repair of X-ray induced DNA strand breaks was impaired despite up-regulation of DNA repair genes. The distinct phenotype of genome instability, increased heterochromatinisation and (in 30% of the cases futile) up-regulation of DNA repair genes was stably maintained over several cell passages indicating that it represents a feature of geroconversion that is distinct from cellular senescence, as it does not encompass a block of proliferation.

## INTRODUCTION

Many mechanisms of skin aging converge on the dermis, a skin compartment consisting mainly of dermal fibroblasts and surrounding matrix [1]. Dermal fibro-blasts are mostly quiescent cells that are regularly exposed to external noxae such as ultra violet light. Therefore, these cells provide a paradigmatic model of long-term cellular adaptation to exogenous stress. Although human diploid fibroblasts have been a preferred model for *in vitro*-studies of cellular ageing, comparatively little is known about the ageing process of these cells in their physiological tissue environment, i.e. the dermis [2]. Features of dermal fibroblast ageing detected by analysis *in vivo* or *ex vivo* encompass alterations of the cytoskeleton [3–6], incipient or manifest cellular senescence [7–10], epigenetic alterations [10, 11], impaired matrix homeostasis [12–17] and mitochondrial dysfunction [18–20]. Here, we address the question whether this list should also encompass instability of the nuclear genome.

Nuclear genome instability is a ubiquitous hallmark of ageing in many tissues and organismal models [21]. Many features of nuclear genome instability have been demonstrated in dermal fibroblasts subjected to stress-induced or replicative senescence *in vitro*. These encompass enhanced chromosome breakage, telomere shortening, telomere dysfunction, accumulation of DNA damage and increased DNA damage signalling [22–28]. Fibroblasts in the dermis of aged baboons exhibit accumulation of nuclear foci positive for P53 binding-protein 1, which is a chromatin mark of DNA double strand breaks. Many of these foci are co-localised with telomere markers suggesting they indicate telomere dysfunction [8]. However, in the aged human dermis or in dermal fibroblasts isolated from old human donors there is no indication of telomere shortening [29, 30]. On the other hand, we have observed that fibroblasts isolated from the dermis of old human donors exhibit down regulation of a gene cluster associated with gene ontology terms of nucleosome assembly, telomere packaging, chromosome maintenance and the mitotic cell cycle as a major systematic alteration [20].

In summary these observations suggest that nuclear genome instability could be a feature of dermal fibroblasts subjected to ageing *in situ*, even though replicative telomere shortening seems not to play a role in ageing of the dermis [29, 30]. To follow up on this notion, we have studied genome stability of primary human fibroblasts retrieved from the bottom side of female breast of donors aged 20 – 67 years [17, 20, 31]. We have restricted our study to female donors in order to avoid confounding effects of gender on biomarkers related to skin aging [32]. Moreover, a comparably small cohort was studied in order to carry out a broader range of investigations encompassing measurements of (i) chromosome integrity, (ii) base line levels and maximal capacity of DNA damage signalling, (iii) the extent of alkaline DNA unwinding due to spontaneous DNA strand breaks, and (iv) the susceptibility to DNA strand break induction by ionising radiation and the repair kinetics thereof.

## RESULTS AND DISCUSSION

### Age-related regulation of genes associated with genome maintenance, cell cycle and DNA-repair

**Figure 1:**
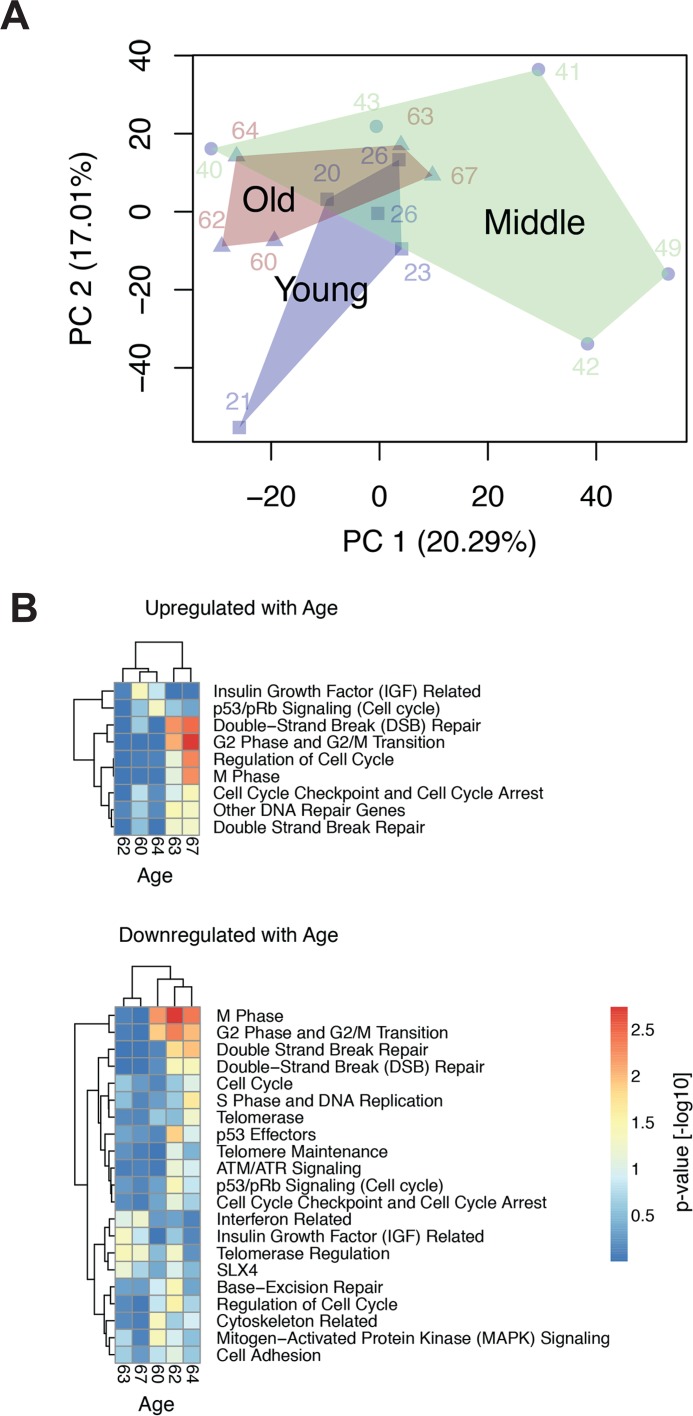
GSEA analysis of age-related regulation of genes associated with genome maintenance (**A**) Principal component analysis of the donor transcriptomes. Samples of the same age group are enclosed by a convex hull to mark the overlap and separation of these groups. (**B**) The heatmaps depict the gene set enrichment analysis of gene sets related to cell cycle, senescence, telomere and DNA repair, which are up- or down-regulated with age (*p*-value < 0.1 in at least one donor). Heatmap colours correspond to the –log_10_ transformed *p*-values. Depicted expression values are row-wise mean centred and scaled to unit variance. Genes and samples (rows and columns, respectively) have been hierarchically clustered using complete linkage. Complete data files of GSEA are provided in [20].

Aging is an inevitable and ubiquitous development in living organisms, while age progression is an individual feature and shows a large heterogeneity among humans. To that effect the fibroblast transcriptomes from the differently aged donor groups studied here hinted at a heterogeneous response as indicated by principal component analysis (Fig. 1A). Pronounced heterogenei-ty of the middle-aged group was previously assigned to the increased body mass index of this group [20]. Here, we show that cells from old donors were also heterogeneous. Some clustered apart from the young donors (samples from donors aged 60, 63 and 67), while others clustered together with the young donors (samples from donors aged 62 and 64). Correspondingly, gene set enrichment analysis (GSEA) on major age-associated pathways revealed a large variation between young and old donor samples. Generally, we observed a down-regulation of gene sets encoding for cell cycle, extracellular matrix and translation [20]. These findings were in part corroborated by corresponding changes in the proteome [6]. To differentiate these diverse functional alterations and identify age-associated transcriptome alterations that are specifically related to genome stability, we here performed a more detailed analysis on gene sets associated with telomere maintenance and -repair, chromosome segregation and -maintenance as well as DNA double-strand repair (See Supplementary Table I). For the old donors that were distinct from the young donors (samples 60, 62 and 64) this analysis suggested an age-related decrease of transcription/translation and cell cycle that was accompanied by impaired telomere and chromosome maintenance (Fig. 1B). However, there was no clear significance for a concordant regulation of DNA repair genes with age. Any expression changes of DNA repair genes in the samples of old donors as compared to young donors appeared to be covariant with up- or down-regulation of cell cycle genes.

### Age-correlated loss of proliferative capacity and increase in heterochromatin marks

**Figure 2:**
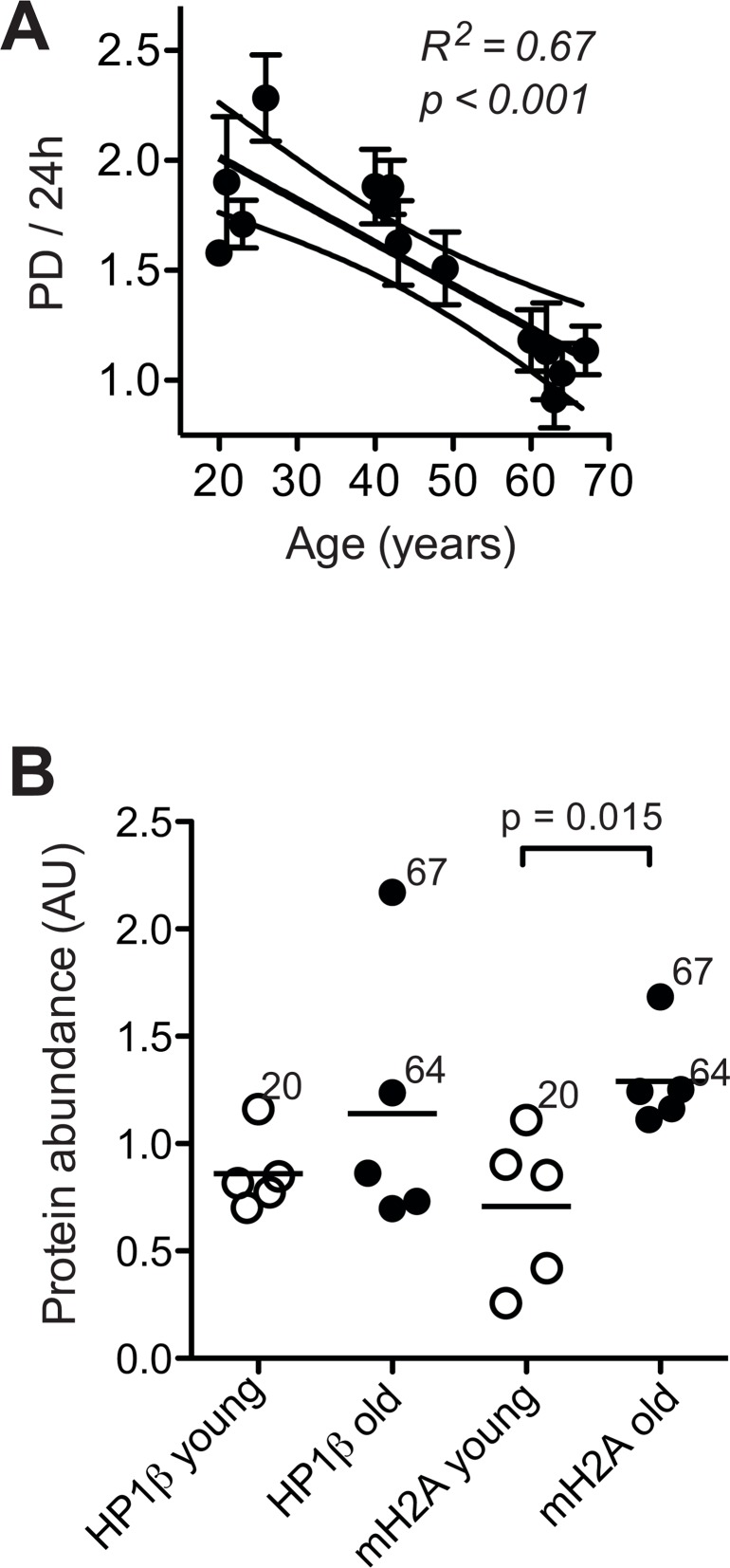
Cell proliferation and heterochromatin marks (**A**) Mean ± SEM of population doublings (PD) per 24 h determined for each donor in five to six independent cultures by seeding a defined amount of cells onto a standardised area of substratum and monitoring the time required for growth to confluency and the final cell yield. Results of linear regression are stated as R^2^: Pearson's coefficient for goodness of fit, *p*: probability for slope = 0, dotted lines: 95% confidence limits of linear regression, and *N.S*.: linear regression of the data revealed no significant age-related change in the parameters. (**B**) Heterochromatin marks HP1β (left) and mH2A (right) determined by immunoblotting in whole cell lysates of dermal fibroblasts from the young (open symbols) and old donor group (closed symbols) at PD < 14. Data were normalised to values obtained in control fibroblasts subjected to replicative senescence. Data points represent means of triplicate determinations in separate cell cultures. Errors were < 30% of the values and are omitted for the sake of clarity. Horizontal lines indicate the mean of the respective age group. Numbers next to data points indicate the chronological age of the respective donor.

It has been pointed out that loss of proliferative capacity is a primordial feature of cell ageing [33]. Indeed, we observed that cell proliferation steadily and significantly decreased with donor age (Fig. 2A), which conforms to age-associated down regulation of genes related to cell cycle progression and mitosis demonstrated in Fig. 1B and Suppl. Tab. 1. The decrease in cell proliferation is not likely related to replicative telomere shortening and replicative senescence, because we have previously shown that replicative cell cycle arrest does not occur below 40 population doublings (PD), and that induction of replicative senescence in culture can be avoided by keeping the cells well below that limit [20]. Moreover, telomere length determined under these conditions exhibited an insignificant decrease of only about 0.01 kb per year of donor age and was on average around 3.6 kilo base pairs (Waldera-Lupa et al., currently under revision in J Invest Dermatol), which is clearly above the limit considered critical for telomere function [34].

Thus, dermal fibroblasts appear to undergo replicative telomere shortening only to a very minor extent during ageing *in situ*, which conforms to current concepts on skin ageing [1, 2]. Consequently, age-related changes of cell functions observed here under these conditions are not likely related to the induction of replicative senescence *in vivo* or *in vitro*. In skin of aged primates heterochromatic marks such as heterochromatin protein 1β (HP1β) and histone macroH2A (mH2A) are increased [10], and human fibroblasts subjected to replicative senescence *in vitro* undergo progressive conversion of eu- into heterochromatin [28]. Here, we observed a significant increase in the expression of mH2A in still replicating fibroblasts from old donors, and a similar trend in the expression of HP1β (Fig. 2B), which indicates that age-related increases in heterochromatin marks can occur independently of replicative senescence. In summary these observations, confirm the concept that cell ageing encompasses two independent processes: loss of proliferative capacity and geroconversion, that is, the acquisition of irreversible age-associated functional alterations such as increased heterochromatinisation [33].

### Increases in chromosome breakage and DNA damage response at base line

Structural and numerical chromosome aberrations are known to increase with age in peripheral nucleated blood cells, buccal epithelia [35–39], hepatocytes [40], vascular smooth muscle cells [41] and human brain [42, 43]. Human fibroblasts subjected in culture to replicative or stress-induced senescence accumulate γH2AX foci [44], chromosome- and centrosome aberrations [24, 26], and exhibit a decline in pathways for rapid repair of DNA double strand breaks (DSB) [25]. A decline of DSB repair capacity was also observed in nucleated peripheral blood cells of old human donors [45]. Based on these reports and in the light of the down regulation of genes associated with genome maintenance in the cell samples studied here (Fig. 1B), we hypothesised that geroconversion occurring in dermal fibroblast during ageing *in situ* could encompass the onset of chromosome instability.

**Figure 3:**
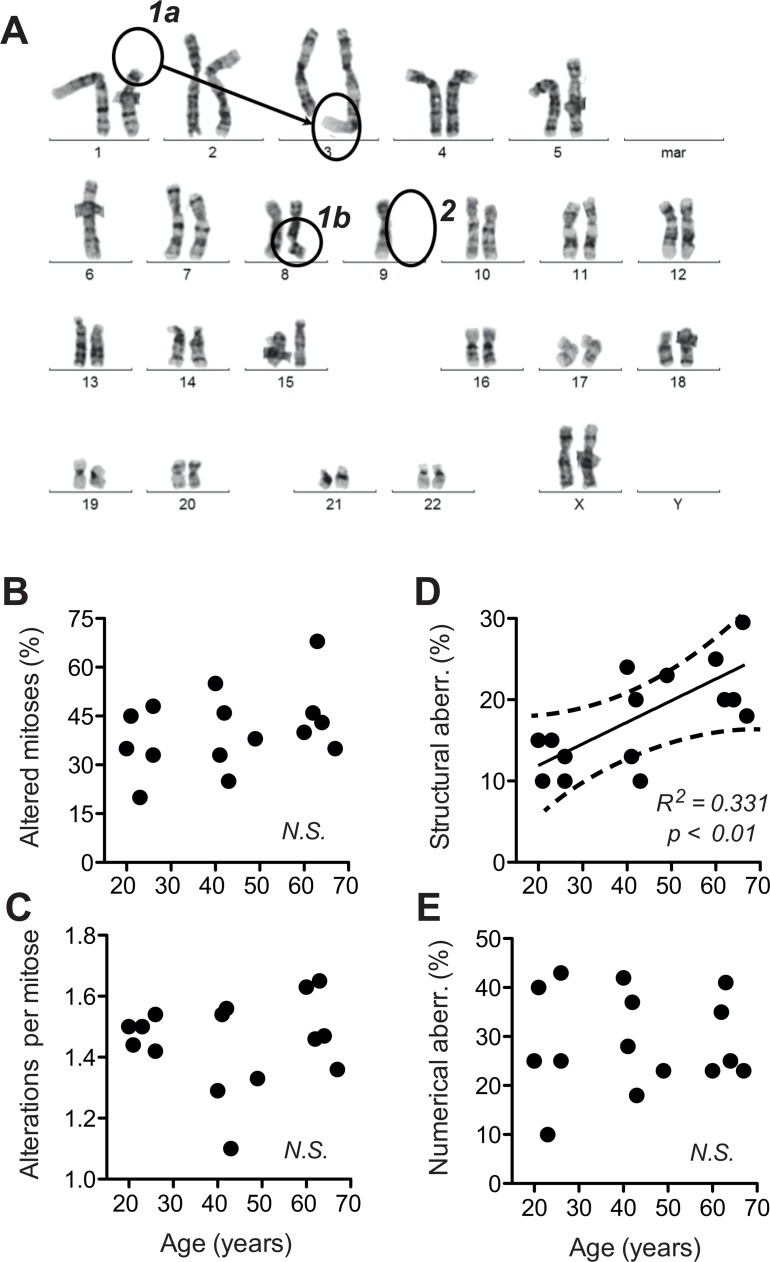
Structural and numerical chromosome aberrations A total of 40 metaphases prepared from two independent cultures were evaluated by G-banding. For a given mitosis, all chromosome aberrations observed therein were listed. (**A**) Examples of structural (1a, b) and numerical (2) chromosome aberrations listed during scoring. (**B**) Frequency of all aberrations evaluated as the percentage of cells (mitoses) positive for one or more aberrations plotted over calendar age of the donors. (**C**) Number of individual aberrations within an altered mitosis plotted over calendar age of the donors. (**D**) Frequency of structural aberrations plotted over donor age. (**E**) Frequency of numerical aberrations plotted over donor age. (**B-E**) Mean values obtained for each donor; SEM < 20% of the values omitted for the sake of clarity; results of linear regression are stated as R^2^: Pearson's coefficient for goodness of fit, p: probability for slope = 0, dotted lines: 95% confidence limits of linear regression, and *N.S*.: linear regression of the data revealed no significant age-related change in the parameters.

To address this question, we subjected the cells to classical cytogenetic analysis. We observed a high incidence of non-clonal chromosome aberrations. About 20% of the mitoses were abnormal and exhibited a variety of abnormalities (Fig. 3B). On average, we found more than one chromosomal aberration per abnormal mitosis (Fig. 3C). The overall frequency of chromosome abnormalities was not sign ificantly correlated to donor age. However, upon differentiation between structural and numerical chromosome aberrations (examples see Fig 3A), we were able to detect a significant age-correlated increase in the incidence of structural aberrations suggesting enhanced chromosome breakage and translocation (Fig. 3D). In contrast, numerical aberrations indicative of mitotic dysfunctions did not increase with donor age (Fig. 3E). These observations suggested that ageing of dermal fibroblasts analysed *ex vivo* involves an increase in chromosome and chromatid breaks.

To address whether the age-related increase in chromosome breaks is associated with an increase in base line levels of DNA doub le-strand breaks (DSB) we quantified histone 2AX phosphorylated at serine 239 (γH2AX), which is a robust quantitative parameter for the DSB-associated DNA damage response (DDR) [46].

**Figure 4:**
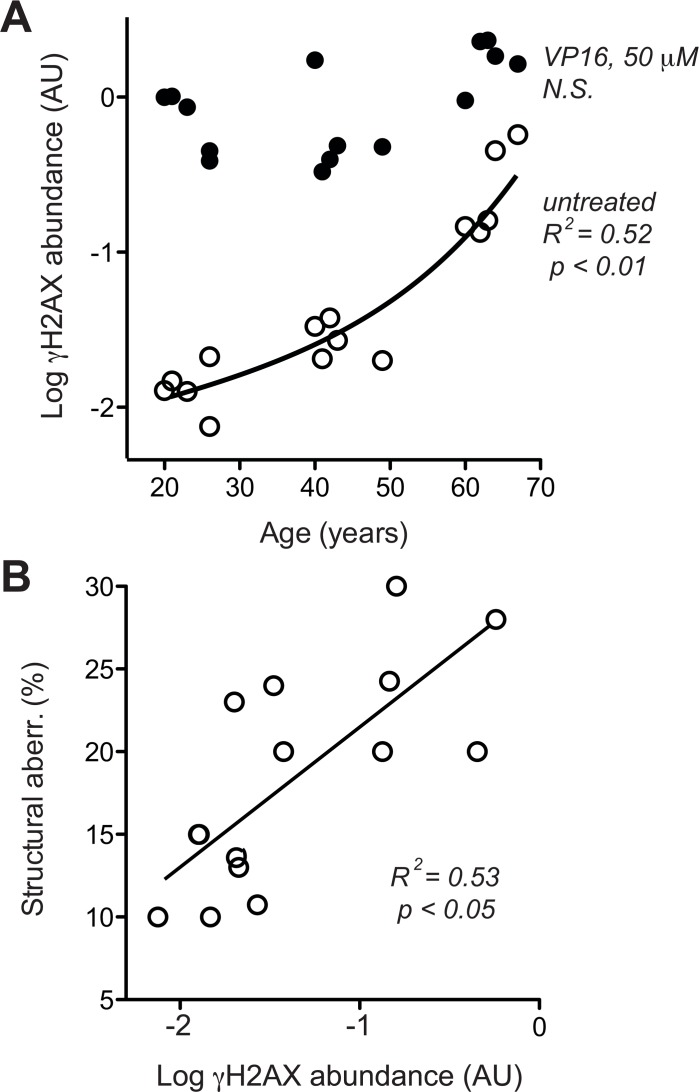
DSB-related DDR **(A**) H2AX phosphorylation was determined in dermal fibroblasts by immune blotting [46] without (open circles) and with (closed circles) pre-treatment with 50 μm VP16. Mean data of three independent cultures, SEM < 20% of the mean is omitted for the sake of clarity. (**B**) Cross-comparison of base line H2AX phosphorylation (in the absence of VP16, same data as represented by open symbols in section A of this figure) and frequency of structural chromosome aberrations (same data as shown in Fig. 3 D). (**A, B**) Results of linear regression are stated as R^2^: Pearson's coefficient for goodness of fit, p: probability for slope = 0, dotted lines: 95% confidence limits of linear regression, and *N.S*.: linear regression of the data revealed no significant age-related change in the parameters.

To compare base line level and maximal capacity of DSB-associated DDR, γH2AX was assessed with and without exposure to the radiomimetic drug etoposide (VP16), which was applied at a dose (50 μM) known to saturate DSB-elicited DDR in human cells [46]. Base line levels of γH2AX showed an age-related exponential increase, while the maximal response levels of γH2AX to 50 μM VP16 did not increase accordingly. As a consequence, the amplitude of the γH2AX response to VP16 declined from about 100-fold in cells from donors aged 20–30 years to less than 10-fold in cells from donors aged 60–70 years (Fig. 4A, please note log scale of y-axis). Moreover, across all cells tested, average base line levels of γH2AX showed a reasonable correlation with the percentage of cells bearing structural chromosome aberrations (Fig. 4B). These data suggest that age-related increases in chromosome breaks could indeed be due to an increase in base line DSB.

### Increased levels of residual DNA breaks at base line

**Figure 5:**
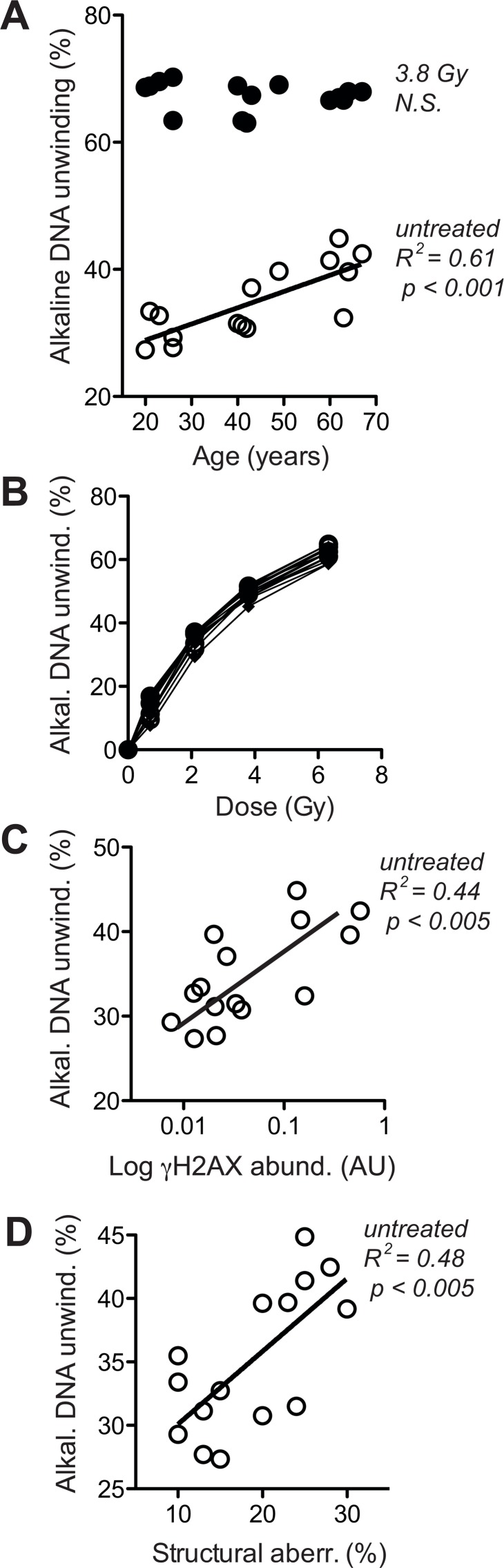
Levels of DNA strand breaks at base line and following IR exposure (**A**) Fluorimetric quantitation of alkaline DNA unwinding carried out with lysates of untreated cells (open circles) and cells exposed to 3.8 Gy (closed circles). Values are normalised to total signal intensity corresponding to non-unwound DNA. (**B**) IR-dose-response curves determined as in (**A**). (**C**) Cross-comparison of base line H2AX phosphorylation (in the absence of VP16, same data as represented by open symbols in Fig. 4A) and base line alkaline DNA unwinding in the absence of IR (same data as open circles in section A of this figure). (**D**) Cross-comparison of structural chromosome aberrations (same data as in Fig. 3D) and base line alkaline DNA unwinding in the absence of IR (same data as open circles in section A of this figure). (**A**, **C**, **D**) Results of linear regression are stated as R^2^: Pearson's coefficient for goodness of fit, p: probability for slope = 0, and *N.S*.: no significant correlation. Data represent mean values of five independent cell cultures per donor, each analysed in four technical replicates. SEM was in all cases less than 20% of the values and is omitted for the sake of clarity.

To corroborate that notion and distinguish whether the age-associated increase in γH2AX (Fig. 4A) was a primary alteration of DDR or a response to increased DSB, we directly assessed overall levels of DNA breaks. This was done by fluorimetric detection of alkaline DNA unwinding (so called FADU-assay) [45, 47, 48], which was measured before and immediately after exposure to ionising radiation (IR, 3.8 Gy). As shown in Fig. 5A, the extent of residual alkaline DNA unwinding before IR exposure increased with age, whereas the extent of additional DNA unwinding induced by IR was the same in cells from old and young donors. The latter observation indicates that the IR-susceptibility of the nuclear genome did not change during ageing, which was confirmed by the stringent overlap of IR dose-response curves of all donors (Fig. 5B). Therefore, the age-related increase in alkaline DNA-unwinding at base line seems due to an increase in residual DSB load unrelated to IR-sensitivity, and that increase could be the reason for the increase in base line γH2AX (Fig. 4A) and the spontaneous increase in chromosome breakage (Fig. 3D). The latter notion is supported by reasonably good correlations between residual alkaline DNA unwinding and base line γH2AX (Fig. 5C) as well as between residual alkaline DNA unwinding and the load of structural chromosome aberrations (Fig. 5D) across cells from all donors. In summary these data strongly suggest that age-correlated increases in base line DNA damage signalling and structural chromosome abnormalities are due to an increase in residual DSB load. However, it remained unclear why residual DSB load increases with donor age.

### Heterogeneous alterations of DSB-repair in old cells

**Figure 6:**
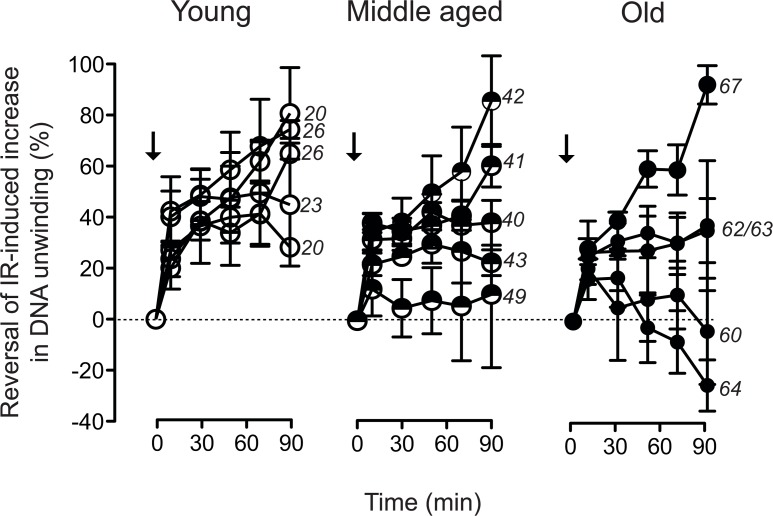
Trajectories of DNA strand break repair Alkaline DNA unwinding was monitored in cell lysates prepared at the indicated time points subsequent to exposure of the cells to 3.8 Gy (arrows). Values are normalised to the amplitude of the initial IR-induced increase in unwinding. Data represent mean values ± SEM of five independent cell cultures analysed for each individual donor. Numbers on the right margins of the age groups indicate the chronological ages of the donors of the samples.

One possible explanation for the observed increase in residual DSB load would be a decrease of repair capacity. However, age-related down regulation of genes involved in telomere maintenance and -repair, chromosome segregation and chromosome maintenance (Fig. 1B), was frequently associated with up regulation of genes involved in DSB repair. This was most conspicuously the case in the samples from donors aged 62/64, which showed reduced expression of genes involved in telomerase regulation and the telomere-associated *SLX4* complex, but at the same time exhibited increased expression of genes playing a prominent role in non-homologous end-joining (NHEJ). This combination is suggestive of a decreased efficiency in chromosome maintenance, which is partially balanced by increased DSB repair by NHEJ. We used the FADU assay to determine the kinetics of disappearance of IR-induced DNA strand breaks, which is considered a valid measure of DSB-repair activity and capacity [45]. Cells were challenged with 3.8 Gy, a dose inducing a half-maximal increase in alkaline DNA unwinding (Fig. 5B). Disappearance of that increase was then monitored over 90 minutes. The data were normalised to the initial IR-induced increase in alkaline DNA unwinding and plotted over time. Fig. 6 demonstrates these repair trajectories for the three age groups. It becomes apparent that the cells from young donors exhibited very similar, mostly overlapping repair trajectories resulting in the disappearance of 40 - 80 % of the IR-induced DNA unwinding (Fig. 6, left, open circles) within 90 min. Repair trajectories of the middle aged donor group (Fig. 6, middle, semi-closed circles) were more heterogeneous with endpoints ranging between 10–90 % disappearance of IR-induced DNA unwinding. Repair trajectories of the old donor group (Fig. 6, right, closed circles) appeared even more heterogeneous: In three specimen repair trajectories were similar as in the middle aged group with endpoints ranging between 40–95 % disappearance of IR-induced DNA unwinding, while the other two specimen exhibited the opposite, namely a progressive increase in DNA unwinding that went beyond the extent initially induced by IR (i.e. an aggravation of DNA damage over time). These results are in line with the gene expression heterogeneity depicted by the PCA and GSEA in Fig. 1: Two samples from old donors (63 and 67) clustered together with the middle aged and young donors. These cells exhibited a higher expression of genes related to cell cycle and DSB repair and had a similar or even better capacity to repair IR-induced DNA damage than the young group. In contrast, the other three specimen of the old group (60,62 and 64), which clustered separately from the young and middle-aged donors, exhibited a decreased expression of genes related to cell cycle and DSB repair, and were less efficient or even unable to repair IR-induced DNA damage (compare Fig. 1A and Fig. 6).

**Figure 7:**
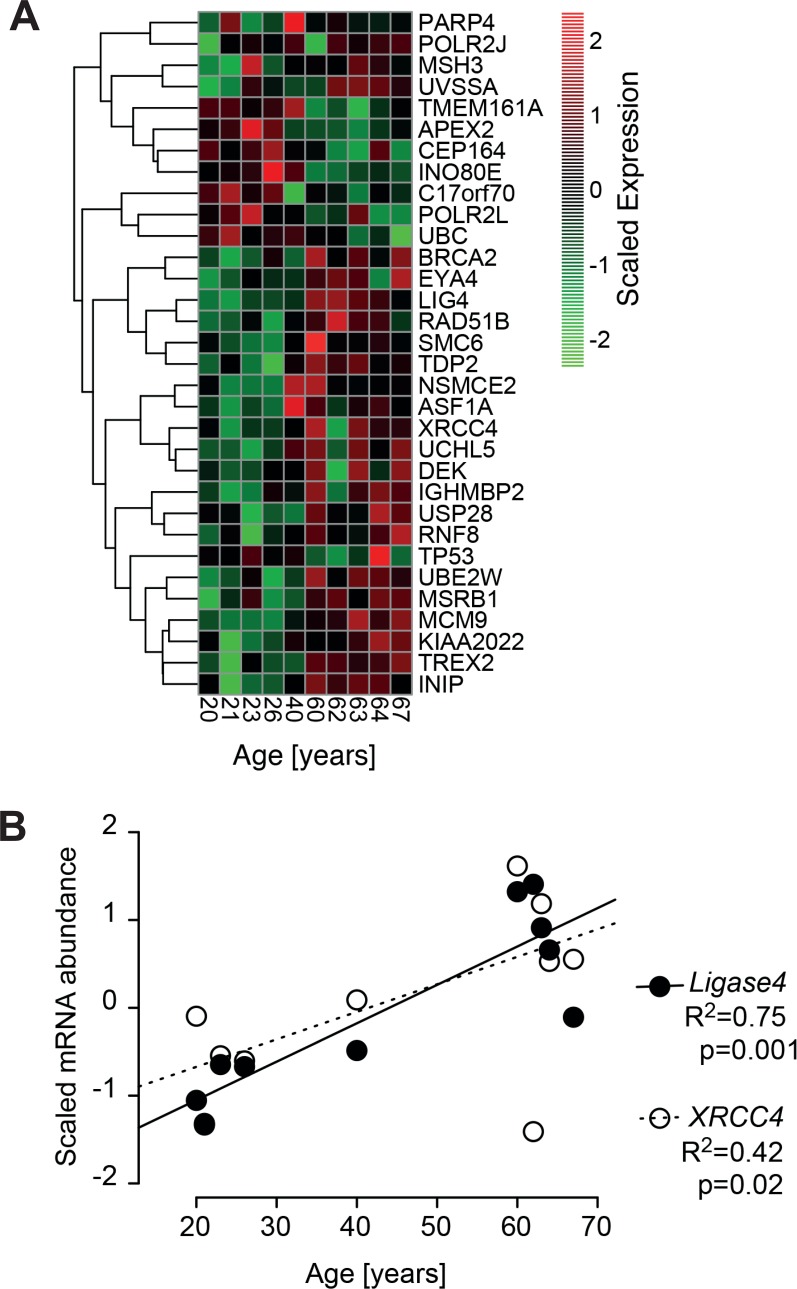
Age-correlated regulation of DNA-repair genes (**A**) Heatmap of significant age-associated changes in genes associated with DNA-repair. The heatmap depicts all genes that are significantly changing with age according to a robust linear regression (*p*-value < 0.1, *R*^2^ > 0.4). The gene list is compiled from 405 genes annotated with DNA repair processes. Depicted expression values are gene-wise mean centred and scaled to unit variance across all samples and hierarchically clustered using complete linkage. Four donors from the middle aged group having a large body mass index and being outliers in the PCA (Fig.1A) have been excluded from the analysis. (**B**) Signal intensities of Agilent array probes representing Ligase 4 (closed circles) and XRCC4 (open circles) plotted over calendar age of the donors. Signal intensities were quantile normalized across all samples and input data were subjected to baseline transformation to the median of all samples. Results of linear regression are stated as *R*^2^: fraction of variance explained by the linear model, *p*: probability for slope = 0.

Thus, in some cases repair of DNA strand breaks became more efficient with age, whereas in other cases it became less efficient or even completely dysfunctional, and it remains unclear what determined this divergent outcome. It seems unlikely that age-correlated regulation of DNA repair genes is primarily involved, since, in line with the repair trajectory experiments (Fig. 6), GSEA revealed a heterogeneous age-related regulation of DNA repair related gene sets (Fig. 1B). Only 32 out of the 405 genes annotated with DNA repair processes were differentially regulated with age (Fig. 7A, robust linear regression. *P*-value < 0.05, *R*^2^ > 0.4). Please note that four donors from the middle aged group having a large body mass index and being outliers in the PCA (Fig.1A) were excluded from that analysis. Among the few repair genes consistently regulated with age, the down-regulated genes (*CEP164* and *TP53*) are also related to the cell cycle. Thus, down regulation is probably associated with the age-related drop in proliferation demonstrated in Fig. 2B. In contrast, age-regulated genes only involved in DSB response and repair (*EYA4, XRCC4* and *LIG4*) were up-regulated with age. Most notably, *XRCC4* and *LIG4* showed a linear, age-correlated increase in expression (Fig. 7B). These two genes code for rate limiting components of NHEJ, the major DSB-repair system available to human dermal fibroblasts at their normal *G*_0_ state [49]. However, despite the uniform, age-correlated up-regulation of these DSB-repair genes, some of the old cells failed in the repair of IR-induced DNA damage, while others excelled.

One possible explanation for the above divergence could be differences in heterochromatin formation. We have demonstrated in Fig. 2A that expression of heterochromatic marks increased with donor age. It is long known that DNA-damage in heterochromatin is refractory to repair [50]. Moreover, primarily un-repairable DSB induce adjacent heterochromatin formation [51]. However, when comparing the expression levels of heterochromatin marks shown in Fig. 2B with the repair trajectories shown in Fig. 6, it turned out that the cells with the highest levels of HP1β and mH2A expression (i.e. donors 20 and 67) exhibited the highest capacity to repair IR-induced DNA damage, which argues against a dampening effect of age-associated heterochromatinisation on the efficiency of these cells to repair IR-induced DNA breaks.

### Concluding remarks

Our data suggest that during ageing *in situ* dermal fibroblast progressively lose their ability to control base line DSB levels, which leads to increased DNA recombination, chromosome breakage and enhanced background noise of DDR signalling. Human fibroblasts subjected to replicative or stress-induced senescence in culture are known to accumulate DNA damage foci believed to represent unrepaired DSB [44] or unrepaired DNA damage at telomeres [27]. Similarly, lymphocytes from aged humans exhibit increases in base line DNA breakage [45, 52, 53] and chromosomal aberrations typically triggered by imprecise repair of DSB via the NHEJ pathway [35, 37]. This type of age-related genome instability is clearly different from the increase in polyploidy observed in aged vascular endothelial cells [54], which is related to telomere shortening and replicative senescence but not to increases in DSB [55]. The question is, whether the age-related increase in DSB observed in fibroblasts and lymphocytes is due to an increased rate of damage or a decreased rate of repair.

There are many indications that age-related increases in residual DSB load and chromosome instability are linked to alterations in DSB repair capacity. NHEJ is probably the only DSB-repair system available to dermal fibroblasts, because in the skin these cells are mostly in *G*_0_ phase and therefore lack a template for repair by homologous recombination [49]. NHEJ becomes less efficient and less precise, when human fibroblasts enter replicative senescence [25], although replicative senescence of dermal fibroblasts induced *in vitro* is not associated with a significant slowing of removal of IR-induced DNA damage [56]. NHEJ activity is also reduced in brains of old rats and Alzheimer disease patients, and it declines in peripheral human lymphocytes with donor age [45, 52, 53, 57]. However, it remains unclear whether age-associated genome instability is the cause or the consequence of age-associated alterations of DSB repair.

Here, we observed that dermal fibroblasts from old human donors have a heterogeneous capacity to remove IR-induced DNA strand breaks. Since IR mostly induces DSB, the observed inter-individual differences in removal of IR-induced DNA damage are most likely due to changes in the efficiency of DSB-repair. Given that gene expression of pace-making elements of the NHEJ pathway showed a uniform age-related increase, the heterogeneous functional outcome of DNA repair possibly indicates the overlay of adaptive and mal-adaptive processes in these long-lived cells. Abrogation of DSB-repair could also be due to accumulation of un-repairable lesions, which has been observed in various other ageing models [27, 44, 58]. A third possibility would be that age-related increases in heterochromatin formation impede DSB-repair in some of the old cells but not in others. However, our data argue against this possibility because all cells showed a similar age-correlated increase in heterochromatin marks. In summary, these considerations prompt the conclusion that the observed alterations of DNA repair capacity reflect a diverse response of age-associated increases in DNA damage and not a cause thereof. In line with that conclusion, a recent systematic analysis of the microRNA-transcriptome of the same cell samples as studied here has revealed a panel of at least 12 microRNAs that exhibit significant age-related up- or down-regulation, but none of the 164 mRNA targets predicted for these microRNAs was related to DNA-repair [6].

Common belief holds that cell cycle arrest is a common feature of senescent and quiescent cells, while cellular senescence emerges from cellular quiescence by the irreversible process of geroconversion, which is driven by *p53*- and *mTOR*-signalling and entails the loss of proliferative potential and the acquisition of cellular hallmarks of aging [33]. A similar increase in DNA damage signalling as seen here in dermal fibroblast from old humans was previously observed in dermal fibroblasts of old baboons and ascribed to the accumulation of senescent cells in the skin of the animals [8]. In our *ex vivo* study we monitored the cells in proliferating culture. Since senescent cells stop to proliferate, they should not accumulate in such a culture and therefore should not contribute significantly to the age-related phenotype changes observed therein. Our data therefore support the alternative concept that in long-lived dermal fibroblasts geroconversion occurring *in vivo* entails the onset of genome-instability as a primary feature, which is stably maintained when the cells are brought back into the cell cycle and therefore unrelated to cellular senescence.

Due to the small cohort size our study is at best a piloting investigation, which, moreover, has been restricted to cells from female donors. Gender and estrogen deficiency at menopause are known to have a much stronger impact on gene expression in skin that chronological age [32]. On the other hand, gender was found to have no influence on DNA damage and DNA repair capacity in human lymphocytes [45]. Thus, it remains an open question whether our results also apply to aging processes in male skin. Clearly, studies on a broader base of biological specimen from both genders are required, to obtain a comprehensive picture of chromosome abnormalities, DNA strand breaks and repair deficiency in dermal human fibroblasts in relation to age.

## METHODS

Donor selection, cell isolation and culture followed published procedures [6, 17, 20, 31]. Briefly, 15 human female donors included in the study were aged 20, 21, 23, 26, 26, 40, 41, 42, 43, 49, 60, 62, 63, 64 and 67 years, thus covering the age spectrum 20 – 67 years and providing five biological replicates for each of the age groups “young” (20–30 years), “middle aged” (40–50 years) and “old” (60–70 years). The body mass index in “young” and “old” groups was ≤ 27 throughout, whereas in the “middle aged” group it ranged from 27 to 31. All donors have given their consent to the study in writing. The investigation conforms to the principles of the Declaration of Helsinki and was approved by the Ethics Committee of the Medical Faculty of the University of Düsseldorf (TOX_EF_Do1/2008). For ethical reasons, the skin samples from which the cells of our study have been cultured were obtained in a completely anonymous form from the department of cosmetic surgery. The only donor-specific data made accessible to us were gender, chronological age, BMI and the skin area from which the cells were isolated. However, given that all samples were obtained through a department exclusively engaged in cosmetic surgery, we can assume that the donors did not suffer from severe health problems such as cancer. A comparably small cohort was studied in order to enable a broad range of analyses. Moreover, our study has been deliberately restricted to female donors in order to minimize variability due to gender influence on skin ageing [32]. All cells were isolated from the same skin area (i.e. the bottom side of female breast) to minimise variances due to body location or different environment exposure. The cells were always studied at a stage of 11 to 13 population doublings, while replicative cell cycle arrest was previously determined to not occur before 40 population doublings [20]. We were thus able to exclude the contribution of replicative telomere shortening to the data. Proliferation rates were determined by seeding defined amounts of cells onto defined areas of substratum and monitoring the times required for growth to confluency and the final cell yield.

Microarray analyses of transcriptomes and biostatistic GSEA analysis of age-related differences in gene expression were done as previously described [20]. Gene sets for analysis were obtained from five RT^2^ RNA QC PCR Array® (SaBiosciences, Qiagen) gene panels for Human Cell Cycle, DNA Damage Signaling Pathway, DNA Repair, Cellular Senescence and Telomeres and Telomerase. The Genes associated with each gene set are listed in Suppl. Table I. Complete data files can be obtained from Gene Expression Omnibus under the submission ID GSE55118.

Cytogenetic analysis. Cells at PD < 14 were grown to 80% coverage of the substratum in DMEM with 2 % glucose and 10 % FBS and treated with colcemid for 6.5 h before harvesting. Preparation of chromosomes was carried out according to standard procedures [59] accredited for clinical diagnostics. For each donor two independent cultures were set up and a total of 40 metaphases were analysed. The evaluation included classical *G*-banding and scoring of chromosome aberrations. All aberrations (gaps, breaks, translocations, deletions, inversions, duplications, rings, derivatives, additions, minutes/double minutes, fragile sites, marker chromosomes) were listed, even when present in a single cell only. The frequency of aberrations was evaluated by counting the percentage of cells (mitoses) positive for one or more of the above aberrations. Losses of single chromosomes were not counted, as these might be preparatory artefacts. Gains of whole chromosomes including marker chromosomes or simultaneous losses of several chromosomes in the same cell were subsumed as numerical aberrations. All other lesions listed above were considered structural aberrations.

Quantitative immunoblotting. To determine hetero-chromatin marks HP1β and mH2A Western blots of whole cell lysates were probed with mouse monoclonal antibodies against HP1β and rabbit polyclonal antibodies against mH2A (Millipore, Schwalbach, Germany). An antibody against β actin (Sigma Aldrich, Munich, Germany) was used to control for loading. Phosphorylated histone 2AX (γH2AX) was assessed by probing Western blots of whole cell lysates with a monoclonal mouse antibody specific for histone 2AX phosphorylated at Ser139. A polyclonal rabbit antibody against total histone 2AX was used to control for loading, as previously described [46]. Both antibodies were obtained from Millipore. Blots were developed with peroxidase-coupled secondary antibodies and chemo-luminescence was quantified with a digital camera system (LAS 4000, Fuji, Dusseldorf, Germany).

DNA strand breaks were measured by automated high throughput fluorimetric detection of alkaline DNA unwinding following published procedures [45, 47, 48]. The loss of fluorescence emission intensity upon alkaline DNA unwinding was used as a measure of overall levels of DNA single plus double strand breaks. This parameter was determined before and immediately after exposure to various doses of ionising radiation (0.7 – 6.33 Gy) to assess base line levels of DNA strand breaks and IR-sensitivity, respectively. To assess capacity and kinetics of DNA strand break repair, alkaline DNA unwinding was monitored for 90 min following exposure to 3.8 Gy. These analyses were carried in lysates from five independent cell cultures of each donor. Each culture was subjected to four replicate measurements.

Statistics of functional data analysis: If not stated otherwise, each variable stated for a given donor and parameter represents mean results from the analysis of three independent primary cell cultures serving as biological replicates. Errors (SEM) refer to the biological replicates and were calculated from the mean of the technical replicates of the assays. Whenever errors are omitted for the sake of clarity they were less than 20% of the values or smaller than the symbols of the data plots. Arbitrary units (AU) derived from relative quantifications (e.g. the densitometry of immunoblots) were normalised to the result obtained with the cells of the youngest donor or to the mean of all values obtained in the same analytical run. GraphPad PRISM 4.0a (GraphPad Software Inc., USA) was used to for linear regression (Pearson), normal data distribution analysis (Shapiro-Wilk) and group comparisons (Wilcoxon's signed rank test or Welch`s unpaired *T*-test). Differences considered statistically significant are marked by * (*p* < 0.05), ** (*p* < 0.01), or *** (*p* < 0.001).

## SUPPLEMENTAL TABLES


