# Supplementary material for: Structural chromosome abnormalities, increased DNA strand breaks and DNA strand break repair deficiency in dermal fibroblasts from old female human donors
Source: Aging (Albany NY). 2015 Feb 4;7(2):110–22. doi: 10.18632/aging.100723 (PMC4359693; doi:10.18632/aging.100723)
Supplement: Supplementary file 1 [file aging-07-0110-s001.pdf]

Supplemental Table I - gene sets related to cell proliferation, cell cycle regulation, genome stability, telomere maintenance and DNA repair that exhibit age-associated alterations in expression

The table below lists the gene sets from Figure 1 for characterization of young and old donors. Gene symbols are listed together with their differential expression between old and young samples according to a moderated t-test. GSEA Significance and direction of gene regulation (up/down: up- down-regulation with age) is given next to each table name on top. The columns for each gene set denote in this order: gene symbol (Symbol), fold change (logFC) between young and old samples (given as log2, a positive (up) value indicates genes that are up-regulated with age), the average expression level (AveExpr, the t-statistic (t), p-value (P.Value), FDR-corrected p-value of differential expression (adj.P.Val) and the Entrez ID (EntrezID) of the genes. The bold titles denote the grouping of the gene sets into Cell Cycle, DNA Damage Signalling Pathway, DNA Repair, Cellular Senescence and Telomeres & Telomerase. The functional gene grouping has been obtained from the Qiagen RT^2 Profile PCR Arrays.

Cell Cycle Gene Sets

Cell Cycle (p=0.2, down)

Symbol	logFC	AveExpr	t	P.Value	adj.P.Val	EntrezID
DDIT3	0.434	9.054	1.934	0.080	0.406	1649
TP53	-0.302	7.379	-2.947	0.014	0.203	7157
MDC1	-0.280	9.258	-2.104	0.060	0.359	9656
CDC25C	-0.255	6.621	-0.774	0.456	0.772	995
CDKN1A	-0.253	10.863	-0.960	0.358	0.714	1026
ATM	-0.242	7.494	-1.586	0.142	0.503	472
PPP1R15A	-0.219	11.211	-0.680	0.511	0.807	23645
CHEK2	0.215	8.078	0.739	0.476	0.785	11200
CDK7	0.145	11.624	0.938	0.369	0.722	1022
ATRIP	-0.134	6.942	-1.531	0.155	0.518	84126
CHEK1	0.093	8.781	1.044	0.320	0.683	1111
MAPK12	-0.087	6.971	-0.687	0.507	0.804	6300
TP73	-0.077	5.554	-0.826	0.427	0.757	7161
PPM1D	-0.049	9.453	-0.256	0.802	0.941	8493
MCPH1	0.047	6.758	0.399	0.698	0.899	79648
ATR	0.047	7.281	0.450	0.662	0.884	545

CDC25A	0.001	6.510	0.005	0.996	0.999	993

G2 Phase and G2/M Transition (p=0.026, down)

Symbol	logFC	AveExpr	t	P.Value	adj.P.Val	EntrezID
GTSE1	-0.634	8.676	-2.074	0.063	0.367	51512
SERTAD1	-0.380	13.001	-2.595	0.026	0.261	29950
BIRC5	-0.352	12.097	-0.810	0.436	0.762	332
CCNB1	-0.303	10.838	-0.890	0.393	0.738	891
ANAPC2	-0.296	7.153	-2.680	0.022	0.243	29882
CDKN3	-0.250	10.359	-0.691	0.505	0.802	1033
CKS1B	-0.195	8.976	-1.017	0.332	0.693	1163
CCNG1	0.194	9.843	1.159	0.272	0.643	900
CCNA2	-0.181	8.265	-0.413	0.688	0.895	890
KPNA2	-0.177	14.245	-1.834	0.095	0.431	3838
CDK5R1	0.148	6.629	1.429	0.182	0.551	8851
CDK7	0.145	11.624	0.938	0.369	0.722	1022
CKS2	-0.142	11.933	-0.521	0.613	0.862	1164
MNAT1	0.063	9.382	0.744	0.473	0.782	4331
CCNT1	0.056	6.219	0.350	0.733	0.916	904
CCNH	0.054	10.813	0.371	0.718	0.909	902
CDK5RAP1	0.050	10.425	0.381	0.710	0.905	51654
BCCIP	-0.003	8.170	-0.020	0.984	0.997	56647
CDC25A	0.001	6.510	0.005	0.996	0.999	993

M Phase (p=0.0089, down)

Symbol	logFC	AveExpr	t	P.Value	adj.P.Val	EntrezID
CDC20	-0.380	9.195	-1.213	0.251	0.626	991
STMN1	-0.368	13.004	-1.483	0.167	0.532	3925
CCNB2	-0.368	11.331	-1.310	0.218	0.591	9133
MRE11A	-0.288	8.199	-2.380	0.037	0.300	4361
CDC25C	-0.255	6.621	-0.774	0.456	0.772	995
AURKB	-0.215	7.289	-0.652	0.528	0.817	9212
CDC6	0.212	5.191	2.543	0.028	0.269	990
CDK1	-0.187	9.027	-0.413	0.688	0.895	983
RAD51	-0.157	8.859	-0.364	0.723	0.911	5888
CCNF	-0.030	5.967	-0.203	0.843	0.952	899
CDC16	0.015	12.812	0.162	0.875	0.963	8881

Cell Cycle Checkpoint and Cell Cycle Arrest (p=0.36, down)

Symbol	logFC	AveExpr	t	P.Value	adj.P.Val	EntrezID
CUL2	0.401	7.869	2.938	0.014	0.204	8453
CDKN2A	-0.324	11.759	-2.028	0.068	0.378	1029
BRCA2	0.320	5.481	1.695	0.119	0.472	675
CDKN2B	-0.304	6.010	-1.645	0.129	0.486	1030

TP53	-0.302	7.379	-2.947	0.014	0.203	7157
CDKN1B	-0.280	9.151	-1.353	0.204	0.575	1027
HUS1	0.271	6.003	2.009	0.071	0.385	3364
CASP3	0.256	11.606	1.833	0.095	0.431	836
CDC25C	-0.255	6.621	-0.774	0.456	0.772	995
CDKN1A	-0.253	10.863	-0.960	0.358	0.714	1026
CDKN3	-0.250	10.359	-0.691	0.505	0.802	1033
ATM	-0.242	7.494	-1.586	0.142	0.503	472
CHEK2	0.215	8.078	0.739	0.476	0.785	11200
GADD45A	0.199	11.958	1.718	0.115	0.465	1647
NBN	0.188	8.270	2.225	0.049	0.335	4683
CDK1	-0.187	9.027	-0.413	0.688	0.895	983
CCNA2	-0.181	8.265	-0.413	0.688	0.895	890
RBBP8	0.171	9.044	1.060	0.313	0.677	5932
CUL3	0.164	5.733	0.939	0.369	0.722	8452
MDM2	-0.157	6.830	-1.019	0.331	0.692	4193
RAD17	0.142	7.047	1.715	0.115	0.465	5884
RAD1	0.112	9.065	0.543	0.599	0.854	5810
CHEK1	0.093	8.781	1.044	0.320	0.683	1111
MAD2L1	-0.092	8.965	-0.269	0.793	0.938	4085
CDC34	-0.079	11.164	-0.678	0.512	0.808	997
CCNG2	-0.078	8.272	-0.331	0.747	0.922	901
CUL1	0.065	9.640	0.467	0.650	0.879	8454
RB1	0.054	10.679	0.559	0.588	0.848	5925
ATR	0.047	7.281	0.450	0.662	0.884	545
KNTC1	-0.037	7.726	-0.200	0.845	0.953	9735
RAD9A	-0.033	7.627	-0.238	0.817	0.944	5883
CDK2	-0.024	6.399	-0.164	0.873	0.963	1017
BRCA1	0.022	7.535	0.088	0.931	0.982	672
WEE1	0.018	10.334	0.112	0.913	0.976	7465
MAD2L2	0.008	10.288	0.087	0.932	0.982	10459
CDC25A	0.001	6.510	0.005	0.996	0.999	993

Regulation of Cell Cycle (p=0.179, down)

Symbol	logFC	AveExpr	t	P.Value	adj.P.Val	EntrezID
CDC20	-0.380	9.195	-1.213	0.251	0.626	991
CCNB2	-0.368	11.331	-1.310	0.218	0.591	9133
AURKA	-0.327	9.192	-1.189	0.260	0.634	6790
BRCA2	0.320	5.481	1.695	0.119	0.472	675
CCNB1	-0.303	10.838	-0.890	0.393	0.738	891
ANAPC2	-0.296	7.153	-2.680	0.022	0.243	29882
CDKN1B	-0.280	9.151	-1.353	0.204	0.575	1027
CCNC	0.267	9.042	2.025	0.069	0.379	892
E2F1	-0.256	8.116	-0.791	0.446	0.768	1869
CDC25C	-0.255	6.621	-0.774	0.456	0.772	995

CDKN1A	-0.253	10.863	-0.960	0.358	0.714	1026
CDK8	0.252	7.825	1.423	0.183	0.553	1024
E2F4	-0.214	9.283	-1.657	0.127	0.481	1874
CDC6	0.212	5.191	2.543	0.028	0.269	990
MKI67	-0.207	8.031	-0.511	0.620	0.866	4288
CCND1	-0.205	11.443	-1.807	0.099	0.437	595
GADD45A	0.199	11.958	1.718	0.115	0.465	1647
CKS1B	-0.195	8.976	-1.017	0.332	0.693	1163
ABL1	0.183	12.176	1.512	0.160	0.524	25
CDK5R1	0.148	6.629	1.429	0.182	0.551	8851
CDK7	0.145	11.624	0.938	0.369	0.722	1022
CDK4	0.138	12.981	0.816	0.432	0.760	1019
CDK6	-0.138	10.748	-0.466	0.651	0.879	1021
CCND2	-0.092	6.624	-0.393	0.702	0.901	894
TFDP2	0.066	9.777	0.519	0.614	0.862	7029
CCNE1	0.063	9.799	0.394	0.701	0.901	898
CCNT1	0.056	6.219	0.350	0.733	0.916	904
CCNH	0.054	10.813	0.371	0.718	0.909	902
RB1	0.054	10.679	0.559	0.588	0.848	5925
BCL2	0.053	7.175	0.375	0.715	0.908	596
ATR	0.047	7.281	0.450	0.662	0.884	545
TFDP1	-0.044	9.112	-0.329	0.748	0.922	7027
KNTC1	-0.037	7.726	-0.200	0.845	0.953	9735
RAD9A	-0.033	7.627	-0.238	0.817	0.944	5883
CCNF	-0.030	5.967	-0.203	0.843	0.952	899
CDK2	-0.024	6.399	-0.164	0.873	0.963	1017
CCND3	-0.023	9.455	-0.196	0.849	0.954	896
SKP2	0.023	6.557	0.113	0.912	0.976	6502
WEE1	0.018	10.334	0.112	0.913	0.976	7465
CDC16	0.015	12.812	0.162	0.875	0.963	8881
BCCIP	-0.003	8.170	-0.020	0.984	0.997	56647

S-Phase and DNA Replication (p=0.12, down)

Symbol	logFC	AveExpr	t	P.Value	adj.P.Val	EntrezID
MCM3	-0.288	11.141	-1.334	0.210	0.584	4172
CDC6	0.212	5.191	2.543	0.028	0.269	990
MCM2	-0.208	9.710	-1.079	0.304	0.671	4171
MCM5	-0.193	9.990	-0.749	0.470	0.781	4174
ABL1	0.183	12.176	1.512	0.160	0.524	25
MCM4	-0.175	8.981	-1.446	0.177	0.546	4173
WEE1	0.018	10.334	0.112	0.913	0.976	7465

DNA Damage Signalling Pathway

ATM/ATR Signalling (p=0.61, down)

Symbol	logFC	AveExpr	t	P.Value	adj.P.Val	EntrezID
SMC1A	-0.526	9.263	-1.655	0.127	0.482	8243
TP53	-0.302	7.379	-2.947	0.014	0.203	7157
MDC1	-0.280	9.258	-2.104	0.060	0.359	9656
FANCD2	0.280	6.192	0.752	0.468	0.780	2177
HUS1	0.271	6.003	2.009	0.071	0.385	3364
ATM	-0.242	7.494	-1.586	0.142	0.503	472
RNF8	0.224	4.950	2.483	0.031	0.278	9025
PARP1	-0.221	11.086	-0.967	0.355	0.713	142
CHEK2	0.215	8.078	0.739	0.476	0.785	11200
RBBP8	0.171	9.044	1.060	0.313	0.677	5932
RAD17	0.142	7.047	1.715	0.115	0.465	5884
ATRIP	-0.134	6.942	-1.531	0.155	0.518	84126
TOPBP1	0.130	8.119	0.832	0.423	0.755	11073
RAD50	0.124	10.130	1.613	0.136	0.495	10111
RAD1	0.112	9.065	0.543	0.599	0.854	5810
BARD1	-0.105	7.900	-0.568	0.582	0.845	580
CSNK2A2	-0.102	9.271	-0.759	0.464	0.778	1459
CHEK1	0.093	8.781	1.044	0.320	0.683	1111
H2AFX	-0.048	11.315	-0.152	0.882	0.965	3014
ATR	0.047	7.281	0.450	0.662	0.884	545
RAD9A	-0.033	7.627	-0.238	0.817	0.944	5883
BRCA1	0.022	7.535	0.088	0.931	0.982	672
RNF168	-0.008	6.567	-0.046	0.964	0.992	165918
CDC25A	0.001	6.510	0.005	0.996	0.999	993

Base-Excision Repair (p=0.088, down)

Symbol	logFC	AveExpr	t	P.Value	adj.P.Val	EntrezID
TP53	-0.302	7.379	-2.947	0.014	0.203	7157
PARP1	-0.221	11.086	-0.967	0.355	0.713	142
NTHL1	-0.199	10.194	-2.241	0.047	0.331	4913
XRCC1	-0.186	11.851	-1.431	0.181	0.551	7515
LIG1	-0.145	9.720	-0.914	0.381	0.731	3978
PCNA	0.141	10.908	1.029	0.326	0.688	5111
FEN1	-0.086	7.757	-0.352	0.732	0.915	2237
OGG1	-0.074	8.552	-0.445	0.665	0.886	4968
MBD4	0.046	8.682	0.424	0.680	0.892	8930
APEX1	0.037	9.414	0.228	0.824	0.947	328
MPG	0.033	12.793	0.281	0.784	0.935	4350
UNG	-0.028	12.474	-0.159	0.876	0.964	7374

Double Strand Break (DSB) Repair (p=0.43, up)

Symbol	logFC	AveExpr	t	P.Value	adj.P.Val	EntrezID
MRE11A	-0.288	8.199	-2.380	0.037	0.300	4361
MDC1	-0.280	9.258	-2.104	0.060	0.359	9656
HUS1	0.271	6.003	2.009	0.071	0.385	3364
RPA1	-0.271	9.125	-0.427	0.678	0.891	6117
ATM	-0.242	7.494	-1.586	0.142	0.503	472
PRKDC	0.215	9.444	0.518	0.615	0.863	5591
NBN	0.188	8.270	2.225	0.049	0.335	4683
RAD51	-0.157	8.859	-0.364	0.723	0.911	5888
LIG1	-0.145	9.720	-0.914	0.381	0.731	3978
RAD50	0.124	10.130	1.613	0.136	0.495	10111
BLM	0.103	6.447	0.333	0.746	0.921	641
XRCC2	0.095	5.728	0.967	0.355	0.713	7516
CHEK1	0.093	8.781	1.044	0.320	0.683	1111
TP53BP1	-0.057	10.951	-0.377	0.714	0.907	7158
H2AFX	-0.048	11.315	-0.152	0.882	0.965	3014
MLH1	0.034	12.257	0.278	0.786	0.936	4292
XRCC6	-0.023	15.827	-0.234	0.819	0.945	2547
BRCA1	0.022	7.535	0.088	0.931	0.982	672

Other DNA Repair Genes (p=0.19, up)

Symbol	logFC	AveExpr	t	P.Value	adj.P.Val	EntrezID
SMC1A	-0.526	9.263	-1.655	0.127	0.482	8243
XRCC3	-0.300	8.158	-2.997	0.013	0.196	7517
CRY1	0.285	6.939	2.330	0.041	0.312	1407
FANCD2	0.280	6.192	0.752	0.468	0.780	2177
RAD51B	0.264	6.514	2.997	0.013	0.196	5890
RNF8	0.224	4.950	2.483	0.031	0.278	9025
REV1	0.201	7.667	2.288	0.044	0.320	51455
GADD45A	0.199	11.958	1.718	0.115	0.465	1647
RBBP8	0.171	9.044	1.060	0.313	0.677	5932
RAD17	0.142	7.047	1.715	0.115	0.465	5884
ATRIP	-0.134	6.942	-1.531	0.155	0.518	84126
TOPBP1	0.130	8.119	0.832	0.423	0.755	11073
FANCG	-0.128	10.233	-1.141	0.279	0.650	2189
FANCA	-0.125	5.803	-0.530	0.607	0.858	2175
ATRX	0.118	5.580	1.210	0.252	0.627	546
RAD1	0.112	9.065	0.543	0.599	0.854	5810
BARD1	-0.105	7.900	-0.568	0.582	0.845	580
BRIP1	-0.062	5.339	-0.836	0.421	0.753	83990
SUMO1	-0.055	9.326	-0.356	0.729	0.914	7341
ATR	0.047	7.281	0.450	0.662	0.884	545
RAD18	0.045	7.259	0.273	0.790	0.937	56852
RAD9A	-0.033	7.627	-0.238	0.817	0.944	5883
CIB1	0.032	12.233	0.309	0.763	0.928	10519
GADD45G	-0.030	7.770	-0.086	0.933	0.983	10912

RAD21	0.027	12.217	0.224	0.827	0.948	5885
RNF168	-0.008	6.567	-0.046	0.964	0.992	165918

DNA Repair

Double-Strand Break Repair (p=0.44, down)

Symbol	logFC	AveExpr	t	P.Value	adj.P.Val	EntrezID
LIG4	0.521	5.284	4.668	0.001	0.066	3981
RAD54L	-0.447	7.677	-0.927	0.375	0.726	8438
BRCA2	0.320	5.481	1.695	0.119	0.472	675
XRCC4	0.309	7.932	2.945	0.014	0.203	7518
XRCC3	-0.300	8.158	-2.997	0.013	0.196	7517
MRE11A	-0.288	8.199	-2.380	0.037	0.300	4361
PRKDC	0.215	9.444	0.518	0.615	0.863	5591
RAD51	-0.157	8.859	-0.364	0.723	0.911	5888
RAD51C	0.154	9.628	1.574	0.145	0.508	5889
RAD52	-0.142	5.816	-1.007	0.336	0.697	5893
RAD50	0.124	10.130	1.613	0.136	0.495	10111
XRCC2	0.095	5.728	0.967	0.355	0.713	7516
XRCC5	0.093	11.382	0.754	0.467	0.780	7520
FEN1	-0.086	7.757	-0.352	0.732	0.915	2237
DMC1	0.046	5.734	0.307	0.765	0.929	11144
RAD21	0.027	12.217	0.224	0.827	0.948	5885
XRCC6	-0.023	15.827	-0.234	0.819	0.945	2547
BRCA1	0.022	7.535	0.088	0.931	0.982	672

Cellular Senescence

p53/pRb Signaling (Cell cycle) (p=0.41, up)

Symbol	logFC	AveExpr	t	P.Value	adj.P.Val	EntrezID
SERPINE1	-1.025	9.391	-3.177	0.009	0.172	5054
CREG1	0.794	12.706	2.933	0.014	0.204	8804
ALDH1A3	-0.674	12.202	-2.587	0.026	0.263	220
SIRT1	0.456	8.583	2.855	0.016	0.216	23411
TGFB1	-0.454	10.331	-2.702	0.021	0.239	7040
ID1	-0.371	13.136	-1.246	0.240	0.615	3397
CITED2	-0.366	13.484	-2.300	0.043	0.318	10370
CDKN2C	0.309	12.062	1.817	0.098	0.435	1031
CCNB1	-0.303	10.838	-0.890	0.393	0.738	891
CDC25C	-0.255	6.621	-0.774	0.456	0.772	995
RBL1	0.251	5.323	1.505	0.162	0.526	5933
PIK3CA	0.212	8.622	1.905	0.084	0.413	5290
ABL1	0.183	12.176	1.512	0.160	0.524	25
CCNA2	-0.181	8.265	-0.413	0.688	0.895	890
MAPK14	-0.167	8.173	-1.638	0.131	0.487	1432

PCNA	0.141	10.908	1.029	0.326	0.688	5111
PLAU	-0.140	9.620	-1.186	0.262	0.635	5328
MAP2K6	-0.114	8.515	-0.460	0.655	0.880	5608
SPARC	0.103	15.565	0.679	0.512	0.807	6678
IGF1R	0.102	10.639	0.510	0.621	0.866	3480
GSK3B	0.083	10.725	0.894	0.391	0.736	2932
MYC	0.074	11.691	0.208	0.840	0.951	4609
AKT1	-0.047	11.221	-0.539	0.601	0.855	207
IGFBP3	-0.044	15.186	-0.157	0.878	0.965	3486
ING1	0.044	8.284	0.497	0.629	0.870	3621
CDKN1C	-0.042	11.376	-0.149	0.884	0.966	1028
MORC3	-0.030	10.053	-0.192	0.851	0.954	23515
SERPINB2	-0.002	6.631	-0.007	0.994	0.999	5055

Interferon Related (p=0.19, down)

Symbol	logFC	AveExpr	t	P.Value	adj.P.Val	EntrezID
ALDH1A3	-0.674	12.202	-2.587	0.026	0.263	220
IRF5	0.349	6.371	3.477	0.005	0.143	3663
IRF3	-0.315	9.395	-1.560	0.148	0.510	3661
IGFBP7	0.313	12.532	0.594	0.565	0.836	3490
CDKN1B	-0.280	9.151	-1.353	0.204	0.575	1027
CDKN1A	-0.253	10.863	-0.960	0.358	0.714	1026
NFKB1	0.117	10.669	1.019	0.331	0.692	4790
IRF7	0.090	10.310	0.576	0.577	0.843	3665
EGR1	-0.066	14.353	-0.233	0.820	0.945	1958
RB1	0.054	10.679	0.559	0.588	0.848	5925
IGFBP3	-0.044	15.186	-0.157	0.878	0.965	3486
IFNG	-0.040	4.957	-0.524	0.611	0.860	3458
SERPINB2	-0.002	6.631	-0.007	0.994	0.999	5055

Insulin Growth Factor (IGF) Related (p=0.45, down)

Symbol	logFC	AveExpr	t	P.Value	adj.P.Val	EntrezID
IGF1	-0.441	5.697	-1.680	0.122	0.475	3479
IGFBP5	-0.382	12.356	-0.577	0.576	0.843	3488
IGFBP7	0.313	12.532	0.594	0.565	0.836	3490
IGF1R	0.102	10.639	0.510	0.621	0.866	3480
IGFBP3	-0.044	15.186	-0.157	0.878	0.965	3486

Mitogen-Activated Protein Kinase (MAPK) Signaling (p=0.05, down)

Symbol	logFC	AveExpr	t	P.Value	adj.P.Val	EntrezID
MAP2K3	-0.276	8.945	-1.407	0.188	0.560	5606
HRAS	-0.178	10.665	-2.128	0.058	0.353	3265
MAPK14	-0.167	8.173	-1.638	0.131	0.487	1432
MAP2K1	-0.155	10.701	-1.219	0.249	0.624	5604
MAP2K6	-0.114	8.515	-0.460	0.655	0.880	5608

Cytoskeleton Related (p=0.16, down)

Symbol	logFC	AveExpr	t	P.Value	adj.P.Val	EntrezID

SERPINE1	-1.025	9.391	-3.177	0.009	0.172	5054
FN1	-0.507	11.875	-1.607	0.137	0.497	2335
PIK3CA	0.212	8.622	1.905	0.084	0.413	5290
HRAS	-0.178	10.665	-2.128	0.058	0.353	3265
PLAU	-0.140	9.620	-1.186	0.262	0.635	5328
SPARC	0.103	15.565	0.679	0.512	0.807	6678
THBS1	-0.074	10.110	-0.156	0.879	0.965	7057
VIM	0.061	17.423	0.867	0.405	0.745	7431
AKT1	-0.047	11.221	-0.539	0.601	0.855	207

Cell Adhesion (p=0.049, down)

Symbol	logFC	AveExpr	t	P.Value	adj.P.Val	EntrezID
TGFB1	-0.454	10.331	-2.702	0.021	0.239	7040
COL1A1	-0.212	16.113	-1.023	0.329	0.691	1277
TGFB1I1	-0.142	13.474	-1.389	0.193	0.564	7041
THBS1	-0.074	10.110	-0.156	0.879	0.965	7057
COL3A1	-0.059	12.092	-0.225	0.826	0.947	1281
CD44	0.013	16.161	0.082	0.936	0.984	960

P53 Effectors (p=0.10, down)

Symbol	logFC	AveExpr	t	P.Value	adj.P.Val	EntrezID
ALDH1A3	-0.674	12.202	-2.587	0.026	0.263	220
E2F1	-0.256	8.116	-0.791	0.446	0.768	1869
HRAS	-0.178	10.665	-2.128	0.058	0.353	3265
MYC	0.074	11.691	0.208	0.840	0.951	4609
IGFBP3	-0.044	15.186	-0.157	0.878	0.965	3486

Telomeres & Telomerase

Telomere Maintenance (p=0.75, down)

Symbol	logFC	AveExpr	t	P.Value	adj.P.Val	EntrezID
POT1	1.013	10.176	7.005	0.000	0.025	25913
PTGES3	0.537	9.254	6.367	0.000	0.033	10728
ERCC1	-0.316	12.162	-2.051	0.066	0.373	2067
SMG6	-0.313	9.606	-1.484	0.167	0.532	23293
MRE11A	-0.288	8.199	-2.380	0.037	0.300	4361
TERF1	0.285	8.390	1.325	0.213	0.587	7013
ACD	-0.230	11.327	-3.126	0.010	0.178	65057
ERCC4	0.225	6.305	1.578	0.144	0.507	2072
PARP1	-0.221	11.086	-0.967	0.355	0.713	142
PRKDC	0.215	9.444	0.518	0.615	0.863	5591
TERT	-0.197	6.018	-2.503	0.030	0.274	7015
NBN	0.188	8.270	2.225	0.049	0.335	4683
DKC1	0.184	10.774	1.152	0.274	0.645	1736
RFC1	0.164	9.979	1.903	0.084	0.413	5981

HSPA1L	-0.162	6.930	-1.795	0.101	0.440	3305
DCLRE1C	-0.150	5.567	-1.573	0.145	0.508	64421
RAD50	0.124	10.130	1.613	0.136	0.495	10111
TNKS2	0.120	8.398	0.916	0.380	0.730	80351
TERF2IP	-0.104	11.740	-1.409	0.188	0.558	54386
BLM	0.103	6.447	0.333	0.746	0.921	641
TERF2	-0.102	7.958	-0.563	0.585	0.847	7014
XRCC5	0.093	11.382	0.754	0.467	0.780	7520
MYC	0.074	11.691	0.208	0.840	0.951	4609
TINF2	-0.050	12.582	-0.550	0.594	0.851	26277
TEP1	0.037	9.066	0.144	0.888	0.967	7011
XRCC6	-0.023	15.827	-0.234	0.819	0.945	2547
TNKS	-0.014	8.717	-0.111	0.914	0.976	8658
PIF1	0.011	8.871	0.129	0.900	0.971	80119
RTEL1	0.005	8.552	0.030	0.977	0.994	51750

Telomerase (p=0.24, down)

Symbol	logFC	AveExpr	t	P.Value	adj.P.Val	EntrezID
TERT	-0.197	6.018	-2.503	0.030	0.274	7015
DKC1	0.184	10.774	1.152	0.274	0.645	1736
NHP2	-0.172	13.821	-1.111	0.291	0.661	55651
WRAP53	-0.150	5.025	-1.182	0.263	0.636	55135
NOP10	0.135	14.397	1.279	0.228	0.602	55505
GAR1	-0.073	12.351	-0.664	0.521	0.813	54433

Telomere-Associated SLX4 Complex (p=0.028, down)

Symbol	logFC	AveExpr	t	P.Value	adj.P.Val	EntrezID
PLK1	-0.707	7.722	-2.732	0.020	0.235	5347
MSH2	-0.370	9.336	-1.952	0.078	0.401	4436
ERCC1	-0.316	12.162	-2.051	0.066	0.373	2067
ERCC4	0.225	6.305	1.578	0.144	0.507	2072
EME1	-0.205	6.136	-1.367	0.200	0.571	146956
MUS81	-0.162	10.216	-1.259	0.235	0.610	80198
SLX4	-0.146	8.246	-0.881	0.398	0.740	84464
MSH3	0.138	9.133	1.243	0.241	0.616	4437
TERF2IP	-0.104	11.740	-1.409	0.188	0.558	54386
TERF2	-0.102	7.958	-0.563	0.585	0.847	7014

Telomerase Regulation (p=0.01, down)

Symbol	logFC	AveExpr	t	P.Value	adj.P.Val	EntrezID
PAX8	-0.474	7.112	-1.068	0.309	0.675	7849
TGFB1	-0.454	10.331	-2.702	0.021	0.239	7040
IGF1	-0.441	5.697	-1.680	0.122	0.475	3479
TP53	-0.302	7.379	-2.947	0.014	0.203	7157
MEN1	-0.265	9.520	-2.585	0.026	0.263	4221

SART1	-0.215	7.489	-1.511	0.160	0.524	9092
PPARG	-0.208	9.615	-0.615	0.551	0.829	5468
PPP2R1B	-0.204	5.002	-2.277	0.045	0.322	5519
ABL1	0.183	12.176	1.512	0.160	0.524	25
SMAD3	-0.149	11.011	-0.788	0.448	0.768	4088
PINX1	-0.137	8.256	-1.352	0.204	0.576	54984
SSB	-0.093	12.132	-0.682	0.510	0.806	6741
EGF	-0.081	5.208	-0.921	0.378	0.729	1950
MYC	0.074	11.691	0.208	0.840	0.951	4609
KRAS	0.063	7.556	0.306	0.765	0.929	3845
ATP5C1	-0.057	11.632	-0.656	0.526	0.816	509
RB1	0.054	10.679	0.559	0.588	0.848	5925
BCL2	0.053	7.175	0.375	0.715	0.908	596
AKT1	-0.047	11.221	-0.539	0.601	0.855	207
SP1	-0.040	9.776	-0.388	0.705	0.903	6667
PRKCB	0.039	4.956	0.228	0.824	0.946	5579
PRKCA	-0.037	11.787	-0.118	0.908	0.974	5578
PPP2R1A	0.020	11.960	0.107	0.917	0.977	5518
